# Protection of infant mice against pertussis, tuberculosis and influenza by co-administration of nasal pertussis vaccine candidate BPZE1 and BCG

**DOI:** 10.1016/j.isci.2025.112839

**Published:** 2025-06-07

**Authors:** Carine Rouanet, Anne-Sophie Debrie, Stephane Cauchi, Nathalie Mielcarek

**Affiliations:** 1 University of Lille, CNRS, Inserm, CHU Lille, Institut Pasteur Lille, U1019-UMR 9017-CIIL-Center for Infection and Immunity of Lille, 59000 Lille, France

**Keywords:** Immunology, Preventive medicine, Disease

## Abstract

Protecting neonates at risk of serious pertussis disease or death represents a global emergency. BPZE1 is the most advanced, next-generation pertussis vaccine undergoing clinical evaluation in children and adults. We investigated the feasibility of co-administering BPZE1 and Bacillus Calmette-Guerin (BCG), the most widely used tuberculosis vaccine worldwide. We showed that BPZE1 can be co-administered with BCG without altering its immunogenicity and protective efficacy against *B. pertussis* in infant mice. Conversely, BCG immunogenicity and protective efficacy against *M. tuberculosis* are not affected by BPZE1. Both vaccines induce off-target protection against heterologous infections. In this study, we showed that BPZE1 and BCG alone or in combination induced high levels of protection against influenza challenge in infant mice. In contrast to BCG, the off-target properties of BPZE1 were independent of the age of vaccination. Vaccination with BPZE1 might be considered at the same time as BCG, facilitating its effective implementation in the childhood immunization schedule.

## Highlights

• Similar homologous protection is induced by co-administration of BPZE1 and BCG
• BPZE1 protection against influenza virus is not affected by BCG co-vaccination
• Unlike BCG, BPZE1’s off-target effects are independent of the age of vaccination

## Graphical abstract

**Figure:**
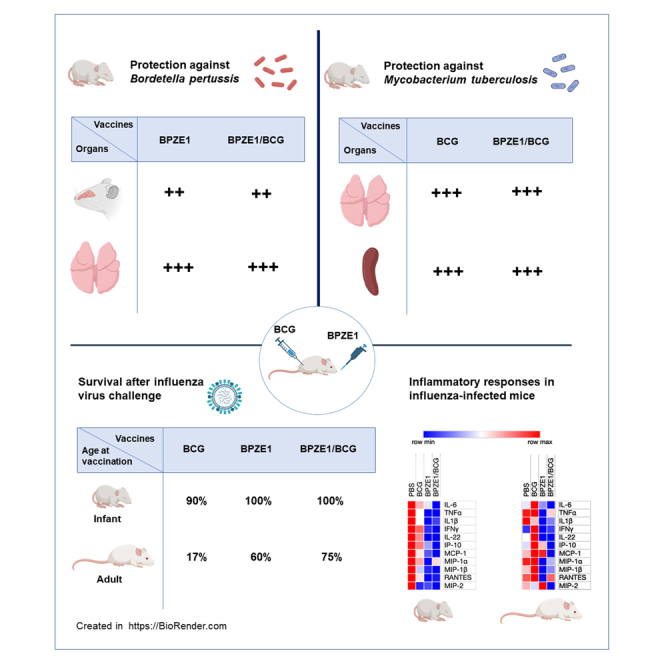

## Introduction

Whooping cough, also known as pertussis, is a highly contagious disease caused by the bacterium *Bordetella pertussis* that affects all ages, but is particularly dangerous for unvaccinated or incompletely vaccinated young infants. An alarming increase in pertussis cases, particularly in infants and adolescents, has been reported in Europe and worldwide in 2023 and 2024.^1^^,^^2^ Despite high vaccine coverage reaching 84% of the world’s population in 2023,^3^ pertussis remains the most poorly controlled vaccine-preventable disease of children worldwide. Toward the end of the 20^th^ century, first generation whole-cell pertussis vaccines were considered too reactogenic and were therefore replaced in many and mostly high-income countries by a second generation of acellular pertussis vaccines (aPv) composed of purified bacterial antigens. This change in the vaccination protocol has been followed by an increased incidence of pertussis disease. Rapid waning of aPv-induced immunity, the inability of aPv’s to induce protective mucosal responses, and to prevent *B. pertussis* colonization and transmission likely explain the current resurgence of pertussis.^4^^,^^5^ To overcome these limitations, we have developed a live attenuated pertussis vaccine given intranasally named BPZE1 to mimic natural infection without pathology.^6^ BPZE1 is a mutated strain of *B. pertussis* that is genetically modified to express an enzymatically inactive form of pertussis toxin and is deficient in the production of the dermonecrotic toxin and the tracheal cytotoxin. Safety, immunogenicity, and protective efficacy of BPZE1 have initially been demonstrated in mice and in non-human primates.^6^^,^^7^^,^^8^ Subsequently, phase 1 and phase 2 clinical trials demonstrated safety and systemic and mucosal immunogenicity of BPZE1 in adults.^9^^,^^10^^,^^11^^,^^12^^,^^13^^,^^14^ A phase 2b study in school-age children aged 6 to 17 years old is on-going in the United Kingdom, Costa Rica and in Australia. While BPZE1 is primarily intended for use as a booster vaccine in school-aged children, adults, and the elderly, the ultimate objective is to vaccinate the most vulnerable population, newborns, against pertussis and this will be studied as part of the pediatric plan to be conducted post-initial licensure.

Simultaneous administration of vaccines, especially at an early age, brings logistical advantages by reducing the number of visits to the pediatrician and may be more acceptable to parents. However, the implementation of such a strategy can only be considered if potential interferences that could reduce the efficacy of co-administered vaccines compared to separate administrations have been assessed and ruled out.

Among the vaccines given to neonates, the Bacillus Calmette-Guerin (BCG) is the most widely used. BCG is the only available vaccine against tuberculosis, a major global public health threat affecting 10.8 million individuals worldwide and resulting in 1.25 million deaths in 2023.^15^ This vaccine is composed of a live attenuated strain of *Mycobacterium bovis*. Administered early in life, BCG is effective in reducing the likelihood and severity of pulmonary, miliary, and meningeal tuberculosis (TB) in infants and young children. Universal BCG vaccination through the WHO’s expanded program on immunization is implemented in 86% of the countries worldwide with more than 100 million newborns vaccinated each year.^16^^,^^17^ BCG vaccination is recommended at birth or in the first week of life. However, there is often a delay in the implementation, as evidenced by recent data indicating that only 37% of the children born in 2016 received early postnatal vaccination, while 90% were vaccinated at one year of age.^16^

Interestingly, both BCG and BPZE1 vaccines have been associated with favorable non-specific properties. A reduction in all-cause mortality has been described in newborns vaccinated with BCG.^18^^,^^19^^,^^20^^,^^21^ The heterologous effects of this vaccine include protection against a large spectrum of viruses, bacteria, and fungi.^22^ In parallel, a large amount of data have been generated in mice regarding the non-specific protective efficacy of BPZE1 against infectious and non-infectious diseases.^23^^,^^24^^,^^25^

In this paper, we used infant mice to assess the feasibility of simultaneous administration of BPZE1 and BCG. The objective was to ascertain that the immunogenicity and protective efficacies of the vaccine candidate BPZE1 against pertussis and BCG against tuberculosis are maintained if the two vaccines are co-administered. We subsequently evaluated the impact of simultaneous immunization on heterologous protection offered by both vaccines using an influenza challenge model.

## Results

### Protective efficacy of BPZE1 against virulent *B. pertussis* infection when co-administered with BCG

**Figure 1:**
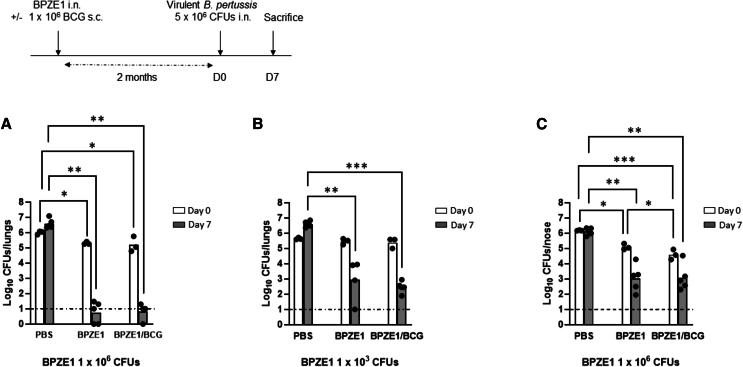
Protection against nasal challenge with virulent *B. pertussis* following co-vaccination with BCG and BPZE1 (A–C) Infant (3-week-old) mice were immunized simultaneously via nasal or subcutaneous route with BPZE1 and BCG, respectively. BPZE1 was administered at either an optimal (A and C) or suboptimal (B) dose for protection. Two months after vaccination, mice were challenged nasally with 5 × 10^6^ CFUs of virulent *B. pertussis* strain BPSM (A and B) or BPCTA1 (C). Bacterial load in the lung (A and B) or in the nose (C) was determined 3h (open columns) or 7 days (filled columns) after challenge. Results are expressed as individual CFU counting and mean from three (day 0) to five (day 7) mice per time point and per group. The dash line corresponds to the limit of detection. A two-factor ART ANOVA, followed by a Conover post hoc test, was conducted to assess statistical significance. *p* values are defined as ≤ 0.05, ∗; ≤0.01, ∗∗; ≤0.001, ∗∗∗.

The potential effect of concomitant BCG vaccination on the protective efficacy of BPZE1 against a virulent *B. pertussis* challenge infection, was evaluated in infant mice (3-week-old) immunized with either 1 × 10^6^ BPZE1 alone intranasally, or simultaneously with 1 × 10^6^ BCG subcutaneously. A group of mice receiving only saline was included as a control. We assessed the safety profile of these vaccination protocols by monitoring the body weight of each group. All mice gained weight during the first two weeks post-immunization (data not shown). Two months after vaccination, animals were challenged intranasally with the virulent *B. pertussis* BPSM strain. The initial BPSM inoculum reaching the lungs was determined 3 h after challenge. Bacterial load in the lungs was subsequently assessed 7 days after infection (Figure 1A). In unvaccinated mice, the colony counts increased by approximately 4-fold between 3 h and 7 days after *B. pertussis* challenge. As previously described,^7^ BPZE1 administration resulted in a near-total protection with CFU counts close to the detection limit (Figure 1A). Comparison between groups immunized with BPZE1 alone or BPZE1/BCG showed that the simultaneous administration of 1 × 10^6^ BCG subcutaneously did not affect the protection provided by BPZE1 (Figure 1A). This led to the hypothesis that the full protection offered by BPZE1 under these conditions might mask the influence of BCG.

The vaccination experiment was therefore repeated with a lower dose of BPZE1 at 1 × 10^3^ CFU (Figure 1B). Under these conditions, vaccination with BPZE1 resulted in a lower but significant level of protection with an approximately 3·5-log reduction in virulent *B. pertussis* load, 7 days post-challenge, compared to unvaccinated animals. Mice vaccinated with either BPZE1 alone or BPZE1/BCG were similarly effectively protected (Figure 1B).

Vaccination with BPZE1 has also been shown to reduce nasopharyngeal colonization by virulent *B. pertussis* in mice and baboons.^8^^,^^26^ The effect of BCG vaccination on the protective efficacy of BPZE1 against nasal colonization by virulent *B. pertussis* was therefore investigated. Infant mice were vaccinated with the optimal dose of BPZE1 and subsequently challenged with the virulent BPCTA1 strain (Figure 1C). This allowed differentiation of BPZE1 persisting in the nasal cavity from the virulent strain used in the challenge infection. Vaccination with BPZE1 resulted in a significant reduction in the number of virulent *B. pertussis* in the nose as early as 3 h after the challenge infection, this reduction becoming even stronger when BPZE1 was administered simultaneously with BCG. Furthermore, the protection against nasal colonization 7 days after the challenge, with a similar 3-log reduction in bacterial load, was as effective in mice vaccinated with BPZE1 alone as with BPZE1 when given with BCG (Figure 1C).

These results demonstrate that BPZE1-induced protection against nasal infection and lung colonization by virulent *B. pertussis* is at least equally effective when BPZE1 is administered alone or concomitantly with BCG.

### Co-administration of BPZE1 and BCG does not alter specific antibody responses

**Figure 2:**
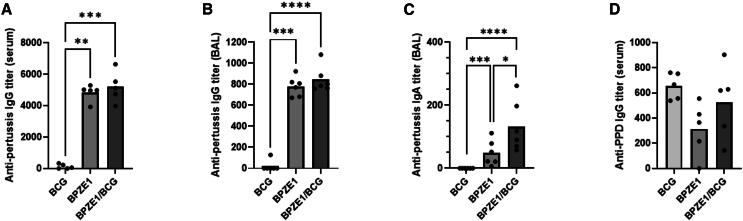
Specific antibody responses after BPZE1 and/or BCG vaccination (A–D) Serum IgG (A and D), BAL IgG (B) and BAL IgA titers (C) were measured against a *B. pertussis* lysate (A, B, and C) or against PPD (D), 21 days after administration of BCG, BPZE1, or both vaccines simultaneously. Results are expressed as individual values and mean for five mice per group. A Kruskal-Wallis test, followed by a Conover post hoc test, was performed to assess statistical significance. *p* values are defined as ≤ 0.05, ∗; ≤0.01, ∗∗; ≤0.001, ∗∗∗; ≤0.0001,∗∗∗∗.

Antibodies contribute to protection against pertussis.^27^ We therefore analyzed the antibody response toward heat-killed *B. pertussis* extract in the BAL fluids and the sera of vaccinated groups, 21 days after immunization (Figures 2A–2C). As anticipated, nasal administration of BPZE1 resulted in the induction of a high level of IgG antibodies, both in the serum (Figure 2A) and in the BAL fluids (Figure 2B) in contrast to the response observed following BCG vaccination. The IgG titers measured in mice vaccinated with both BPZE1 and BCG were comparable to or slightly higher than that observed in the group vaccinated with BPZE1 alone (Figures 2A and 2B). A significant, albeit low, anti-pertussis IgA titer was also measured in the BAL fluids (Figure 2C). This mucosal antibody response was enhanced by a simultaneous BCG vaccination. These results show that co-administration of BCG with BPZE1 does not alter the specific antibody response induced by BPZE1. Conversely, we analyzed the antibody response against purified protein derivative (PPD), which corresponds to the protein fraction of heat-inactivated culture filtrate of *M. tuberculosis* (Figure 2D). No significant difference in serum IgG titer was observed between groups vaccinated with BCG alone or receiving BCG and BPZE1 at the same time. In BAL fluids, IgG and IgA responses to PPD were below the detection threshold of 50.

### Co-vaccination with BPZE1 and BCG induces a positive trend toward a protective cellular response

**Figure 3:**
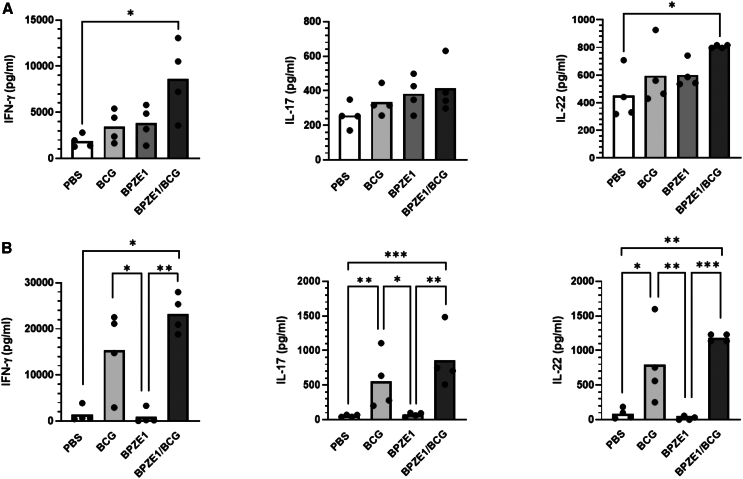
Lung cytokines produced by BPZE1 and/or BCG vaccination (A and B) Infant (3-week-old) mice were immunized with BCG s.c., BPZE1 i.n., or both vaccines simultaneously. Two months after vaccination, mice were sacrificed and pulmonary cells were stimulated with *B. pertussis* lysate (A) or PPD (B). The secretion of IFN-γ, IL-17A, and IL-22 in the culture supernatant was quantified by ELISA. The results are expressed as individual values and mean for four mice per group. A Kruskal-Wallis test followed by a Conover post-test was used to determine statistical significance. *p* values are defined as ≤ 0.05, ∗; ≤0.01, ∗∗; ≤0.001, ∗∗∗.

Vaccine-induced T cell responses play a major role in protective immunity against both pertussis and tuberculosis. We therefore evaluated whether co-vaccination with BPZE1 and BCG modulates the pulmonary cellular immune response compared with administration of each vaccine separately. Two months after immunization, the lungs of the vaccinated animals were recovered and the secretion of IFN-γ, IL-17A, and IL-22 in response to PPD or heat-killed *B. pertussis* extract was quantified (Figure 3). IFN-γ and IL-17A were selected as they have been demonstrated to play a key role in the protection against both *B. pertussis* and *M. tuberculosis*.^27^^,^^28^^,^^29^ IL-22, a member of the IL-10 cytokine family, has been identified as a critical mediator of early mucosal defense and protection against epithelial lung damages.^30^^,^^31^

Two months after vaccination, nasal administration of BPZE1 led to the production of IFN-γ, IL-17A, and IL-22 by pulmonary cells stimulated *in vitro* with a *B. pertussis* extract (Figure 3A). These cytokine levels were comparable to the group of mice vaccinated with BCG alone, suggesting a cross-reactivity phenomenon. Interestingly, the co-administration of BCG with BPZE1 resulted in a non-significant enhancement of *B. pertussis*-specific lung cytokines production compared to the administration of BPZE1 alone. Furthermore, the levels of IFN-γ and IL-22 were significantly higher in co-vaccinated mice compared to unvaccinated controls. The same pattern as in the lung was observed between the different groups for IFN-γ and IL-17 when splenocytes were stimulated *in vitro* with a *B. pertussis* extract (Figure S1A). However, in this case, the specific IL-17 response induced by BPZE1 vaccination was significantly different from that observed in the control and BCG vaccinated groups (Figure S1A). The IL-22 response was not affected by the co-administration of BCG.

We next measured the cytokine levels in response to the stimulation of pulmonary cells with mycobacterial antigens. Almost no secretion of IFN-γ, IL-17A, and IL-22 was observed when cells from BPZE1-vaccinated animals were stimulated with mycobacterial PPD antigens (Figure 3B). In contrast, subcutaneous vaccination with BCG alone induced significant production of PPD-specific IFN-γ, IL-17A, and IL-22 by pulmonary cells. Further analysis revealed an increase, although not statistically significant, of mycobacterial-specific cytokine levels when the two live vaccines were co-administered in comparison to BCG alone (Figure 3B). The specific cytokine response to PPD was also analyzed in the spleen. In this organ, we observed that the simultaneous administration of BPZE1 did not affect the production of any of the cytokines tested when compared to BCG alone (Figure S1B).

### Protective efficacy of BCG against *M. tuberculosis* infection when co-administered with BPZE1

Further studies were conducted to evaluate the protective efficacy of BCG against an *M. tuberculosis* intranasal infection in mice that had been co-vaccinated with BPZE1.

**Figure 4:**
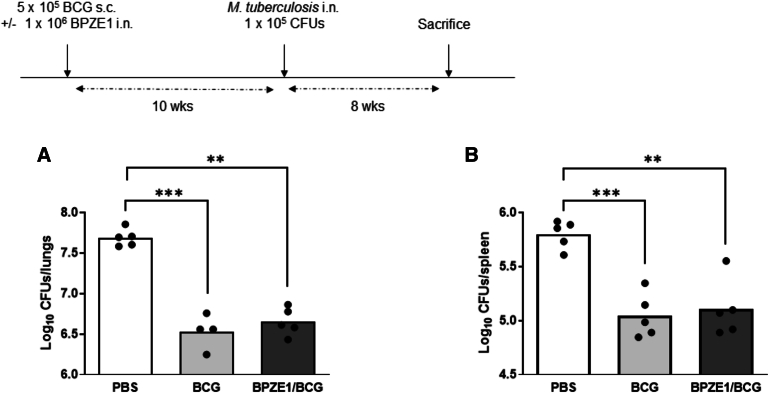
Protection against *M. tuberculosis* in mice co-vaccinated with BCG and BPZE1 (A and B) Infant (3-week-old) mice were immunized s.c. with BCG alone or simultaneously with in. BPZE1. Ten weeks after vaccination, mice were challenged nasally with the virulent *M. tuberculosis* H37Rv strain. Bacterial load in the lungs (A) and spleen (B) was determined 8 weeks after challenge. Results are expressed as individual CFU counting and mean from five mice per group. A Kruskal-Wallis test followed by a Conover post-test was used to determine statistical significance. *p* values are defined as ≤ 0.05, ∗; ≤0.01, ∗∗; ≤0.001, ∗∗∗.

Ten weeks after vaccine administration to 3-week-old infant mice, the animals were challenged intranasally with the virulent *M. tuberculosis* H37Rv strain (Figure 4). Bacterial load in the lungs and spleen was evaluated 8 weeks post-challenge. BCG immunization resulted in a significant decrease in bacterial load with a 1·2-log reduction in the lungs (Figure 4A) and a 0·8-log reduction in the spleen (Figure 4B), when compared to unvaccinated animals. A similar level of protection was observed in the group of mice that received nasal administration of BPZE1 at the time of BCG vaccination (Figure 4).

### BPZE1 protects against influenza regardless of co-administration of BCG and age at vaccination

Live BPZE1 and BCG vaccines have been shown to confer protection against heterologous infections. Notably, it has been demonstrated that a single administration of BPZE1 is capable of protecting mice against the highly pathogenic mouse-adapted H3N2 influenza A virus.^32^ We investigated here the impact of simultaneous BPZE1 and BCG vaccination on influenza-related morbidity and mortality.

Infant (3-week-old) mice were immunized with BPZE1, BCG, or received both vaccines. Six weeks after immunization, the animals were challenged intranasally with 300 pfu of H3N2 mouse-adapted influenza virus. Mice that had not been vaccinated before the challenge served as a control group. The weight and mortality of the animals were monitored daily, and the mice were sacrificed when the body weight loss exceeded 20% of the original body weight.

**Figure 5:**
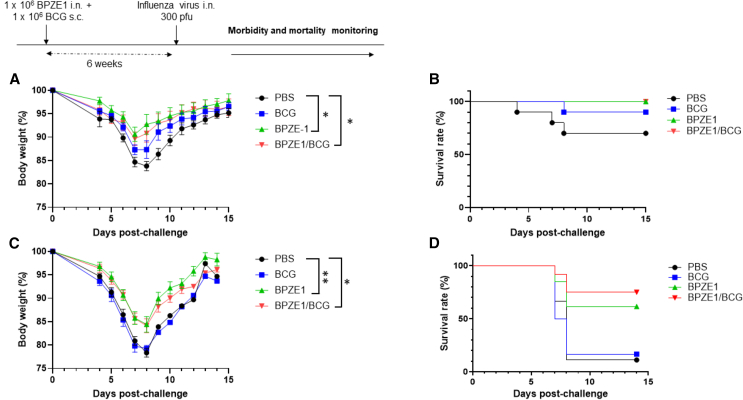
Heterologous protection against lethal challenge with influenza A virus following co-vaccination with BCG and BPZE1 (A–D) Three-week-old infant (A and B) or 8-week-old adult (C and D) mice were immunized with BPZE1 nasally, BCG s.c., or they received both vaccines at the same time. Viral challenge was performed six weeks after vaccination. Six to thirteen mice were included per group. Body weights were monitored daily, and mice were sacrificed when the body weight loss exceeded 20% of initial body weight. Results are expressed as mean ± SEM (A and C). Mixed-effects models were used to compare weight loss over time between different conditions. Survival rate was analyzed for each group (B and D). The log*-*rank test was used for survival analysis. *p* values are defined as ≤ 0.05, ∗; ≤0.01, ∗∗.

Naive infant animals infected with the influenza virus exhibited a rapid reduction in body weight. Notably, immunization with BPZE1 protected infant mice against influenza-induced morbidity as evidenced by a significant reduction in body weight loss compared to non-vaccinated mice (Figure 5A, *p* ≤ 0·05). Interestingly, the same body weight curve was observed for the animals vaccinated simultaneously with BPZE1 and BCG compared to BPZE1 alone. Furthermore, a single dose of BPZE1, whether alone or concomitantly with BCG, demonstrated protective efficacy against mortality with a 100% survival rate recorded in both cases. This compares favorably with the 70% survival rate observed in the non-vaccinated group (Figure 5B). We also observed a protective effect of BCG alone, with a 90% survival rate observed for BCG-vaccinated mice (Figure 5B). However, the reduction in weight loss compared with non-vaccinated animals was less pronounced than with BPZE1 immunization (Figure 5A).

The same experiment was repeated in 8-week-old adult mice, and the results indicated that mice vaccinated with BPZE1 were significantly protected against a challenge with influenza A virus compared to non-vaccinated animals. This was demonstrated by monitoring both body weight changes (Figure 5C) and survival rate (Figure 5D). The *p* values for these comparisons were *p* ≤ 0·01 for body weight and *p* ≤ 0·05 for survival rate. With regard to the mortality rate, this protective effect was even more pronounced when BPZE1 was administered simultaneously with BCG (*p* ≤ 0·01) (Figure 5D). In contrast, no benefit of BCG alone was observed on weight loss or survival rate in adult mice when compared to non-vaccinated animals.

### BPZE1 vaccination, with or without BCG, reduces the inflammatory responses to influenza virus

**Figure 6:**
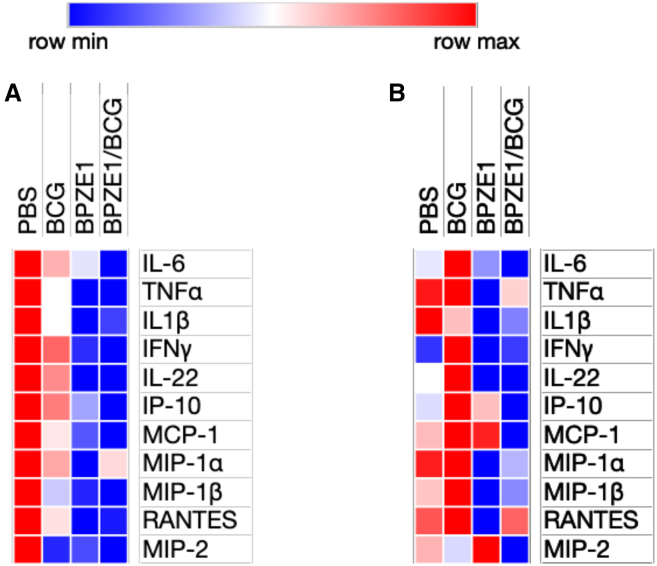
Cytokine and chemokine profile, in murine BAL fluids, 5 days after challenge with influenza A virus (A and B) Three-week-old infant (A) or 8-week-old adult BALB/c (B) mice were vaccinated with BCG s.c., BPZE1 i.n., or both vaccines simultaneously. Viral challenge was performed six weeks after vaccination. Five days after the infection, the animals were sacrificed and BAL fluids were collected. The production of various cytokines and chemokines was quantified by Luminex in the individual BAL fluid samples. Heatmaps were generated to visualize the standardized data. The scaled expression values (row *Z* score) of each cytokine and chemokine are represented using a red-blue color scale. Red indicates higher relative abundance, while blue denotes lower relative abundance.

Both cytokines and chemokines play a key role in the morbidity and mortality associated with influenza infection. While a moderate inflammatory response is protective against the virus, excessive cytokine response and chemokines, such as MCP-1, have been associated with disease severity and mortality in influenza A infection.^33^ BPZE1 has previously been shown to protect mice against influenza A virus mortality by dampening the cytokine storm resulting from the H3N2 infection.^32^ We therefore measured the levels of several cytokines, including the pro-inflammatory TNF, IL-1β, IL-6, and IFN-γ, as well as IL-4, IL-10, IL-17A, and IL-22, in BAL samples from mice 5 days after influenza challenge. The production of IL-4, IL-10, and IL-17A was below the limit of detection regardless of the age of the animals. Z scores were calculated on a gene-by-gene (row-by-row) basis and applied to heat map visualizations, allowing for standardized comparison of cytokine and chemokine expression patterns across samples (Figure 6). For more details, raw data are provided in Figure S2.

Mice vaccinated with BPZE1 as infants showed reduced production of the four pro-inflammatory cytokines TNF, IL-6, IL-1β, and IFN-γ in response to influenza infection compared to unvaccinated animals (Figures 6A and S2A). In adult mice, BPZE1 vaccination reduced TNF and IL-1β production, although not significantly in response to influenza virus compared to unvaccinated animals (Figures 6B and S2B). This supports the notion that BPZE1 can prevent an excessive and harmful inflammatory response regardless of the age at vaccination. In contrast, BCG vaccination alone also resulted in a slightly lower inflammatory response compared to unvaccinated animals, but only if given to infant mice (Figure 6 and S2A). In adult animals, TNF, IL-6, and IL1β production was comparable to that observed in the unvaccinated group, while IFN-γ secretion was higher, albeit not statistically significant (Figure S2B). Notably, the BPZE1-induced reduction of pro-inflammatory cytokine responses was not affected by co-administration of BCG, regardless of the age of the mice. We next analyzed the production of IL-22 in the BAL as this cytokine is known to be produced in the lungs during influenza infection.^34^ Infant mice vaccinated with BPZE1 alone or concomitantly with BCG had significantly lower levels of IL-22 secretion compared to unvaccinated mice or mice vaccinated with BCG alone (Figures 6A and S2A). These data are consistent with the severity of the disease in each of these groups of mice (Figures 5A and 5B).

The production of several chemokines involved in influenza severity,^33^ including CCL2/MCP-1, CCL3/MIP-1α, CCL4/MIP-1β, CCL5/RANTES, CXCL2/MIP-2, and CXCL10/IP-10 was also quantified. In mice vaccinated early in life with BPZE1 and challenged 6 weeks later with influenza virus, a significant reduction in MCP-1 secretion and a decrease, albeit not statistically significant, in all other tested chemokines, was observed (Figures 6A and S2C). Co-administration of BPZE1 and BCG led to a more pronounced reduction in MCP-1 and MIP-2 secretion after influenza challenge. In contrast, vaccination of adult mice had no effect on chemokine production after influenza infection, irrespective of the vaccine(s) administered (Figures 6B and S2D).

These data collectively confirmed that BPZE1 is able to reduce lung inflammation caused by influenza A virus infection, in both young and adult mice. This effect was even more pronounced when co-vaccinated with BCG. In addition, our results suggest that the age at which the vaccination takes place may also influence the level of inflammatory response observed.

## Discussion

This study investigated the impact of co-administering two live bacterial vaccines, BPZE1 and BCG, on the induction of immune responses and the homologous and heterologous protective efficacy of the vaccines. BPZE1 is the most advanced next-generation pertussis vaccine, delivered intranasally and designed to prevent *B. pertussis* nasal infection and disease. BPZE1 has demonstrated safety and immunogenicity in four phase 2 clinical trials in adults and school-aged children and will soon enter phase 3 trials (unpublished data).^13^^,^^14^ Future studies will aim to immunize neonates. In this context, co-administration of BPZE1 with the world’s most widely used vaccine, BCG against tuberculosis, should be considered. Indeed, concomitant administration of two or more vaccines is one of the most effective approaches to increase vaccination coverage, reducing the costs associated with the number of vaccination visits, and reducing the number of missed vaccination opportunities. However, immunological interference may occur between vaccines administered simultaneously, leading to a potentially detrimental reduction in the immunogenicity and protective efficacy of each vaccine. It is therefore an important parameter to assess, particularly when vulnerable individuals such as neonates are the target population.

Protective immunity induced by BPZE1 and BCG against *B. pertussis* and *M. tuberculosis* respectively, has been associated with the induction of cellular Th1/Th17 type cellular immune responses.^6^^,^^35^^,^^36^ Our study showed that the concomitant administration of BPZE1 with BCG positively polarized cellular immunity toward a more pronounced Th1/Th17 subset with an increase, albeit not statistically significant in lungs, in the production of IFN-γ and IL-17A. We also showed that simultaneous vaccination with BPZE1 and BCG induced high levels of IgG responses toward *B. pertussis* antigens, comparable to those induced by BPZE1 alone, and increased IgA secretion. Taken together, these data suggest that BCG does not alter the global immunogenicity of BPZE1. Consistent with these data, we have shown that co-administration of BCG does not impair the protective efficacy against virulent *B. pertussis* infection induced by nasal immunization with BPZE1. The high level of BPZE1-induced protection against *B. pertussis* infection was assessed both in the lungs and in the nasal cavity. The latter point is particularly important as prevention of nasal colonization is a key step in preventing asymptomatic carriage and transmission. Importantly, the immunogenicity and protective efficacy of BCG against a virulent strain of *M*. *tuberculosis* was also fully preserved when administered concomitantly with BPZE1.

The antigen-specific IL-22 response induced by both vaccines was also analyzed. IL-22 has been shown to play a role in mediating protective immunity against *M. tuberculosis* and more specifically, to contribute to BCG-induced protection against *M. tuberculosis*.^37^^,^^38^ The role of IL-22 in protection against *B. pertussis* infection has not yet been elucidated. However, IL-22 has been detected in the blood of individuals who received a booster dose of whole-cell or acellular pertussis vaccine.^39^^,^^40^ In our study, an increase in specific IL-22 release against both *B. pertussis* and *M. tuberculosis* antigens was measured in the lungs, when the two vaccines were administered together. This observation argues for beneficial off-target effects of this double vaccination, as IL-22 is a critical mediator of early mucosal defense and protection against epithelial lung damage.^30^^,^^31^ Like several other live attenuated vaccines including BCG, BPZE1 has been shown to elicit beneficial off-target effects, protecting against heterologous infections and inflammatory diseases.^23^ In mice, BPZE1 has been shown to protect against heterologous respiratory infections common in childhood, including *Streptococcus pneumoniae*,^24^ respiratory syncytial virus,^41^ and influenza A virus.^32^ Similarly, neonatal BCG vaccination has been associated with a decreased all-cause mortality in children under 5 years of age, particularly in high-mortality settings.^42^^,^^43^ In our study, we analyzed the off-target effect of co-vaccination with BPZE1 and BCG on the heterologous protection against a mouse-adapted H3N2 influenza A virus. In infant mice, vaccination with BPZE1 alone or concomitantly with BCG resulted in 100% survival after influenza challenge. This protection against influenza-induced morbidity and mortality was associated with a reduction of inflammatory cytokines as well as MCP-1 and MIP-2 chemokines in BAL fluids compared to those from unvaccinated animals. BCG vaccination alone achieved a similar level of heterologous protection with 90% survival rate. In contrast, in adult mice, the protection against influenza-induced morbidity and mortality was observed only in BPZE1-vaccinated mice and was slightly increased in mice receiving both BPZE1 and BCG. There was no protective effect of BCG alone against influenza challenge in adult mice. This discrepancy between the off-target effect of BCG and the age of the mice at the time of vaccination is consistent with recent clinical studies, which showed that adult vaccination with BCG, in contrast to neonatal vaccination, was associated with a slight increase in the incidence of subsequent febrile or respiratory diseases.^44^ Interestingly, we showed that the off-target protective efficacy of BPZE1 was strong regardless of the age of vaccination, as evidenced by the protection against influenza infection in both infant and adult mice. In addition, BCG vaccination at the same time as BPZE1 did not alter the heterologous protective efficacy of BPZE1.

Further studies are needed to understand the mechanisms induced by BPZE1 that lead to non-specific protection against other pathogens. Trained innate immunity is considered a key mechanism in the off-target protection associated with live vaccines and BCG-induced innate immunity has been extensively studied in this context.^45^ Innate immune cells, namely macrophages, play a key role in this mechanism through the expression of pro-inflammatory cytokines upon stimulation by live vaccines or by pathogens. We have recently investigated the complex regulation of pro-inflammatory cytokines in alveolar macrophages infected with *B. pertussis* and explored the role of the signal transducer and activator of transcription (STAT) protein family in this innate immune response.^46^ Epigenetic and cellular mechanisms involved in the induction of innate immune memory upon interaction of macrophages with BPZE1 or virulent *B. pertussis* will be further investigated.

Pertussis remains endemic despite a high vaccination coverage highlighting the failure of currently available pertussis vaccines to prevent infection and transmission, as well as the rapid waning of vaccine-induced protective immunity.^47^^,^^48^ In 2023–2024, after a reduced pertussis incidence during the COVID-19 pandemic, a sharp increase in pertussis cases has been reported worldwide, further underlining the urgent need to provide the population with a new-generation pertussis vaccine capable of reducing infection and disease.^49^ We have developed a nasal pertussis vaccine candidate, BPZE1, which is in advanced clinical development. The ultimate objective is to vaccinate the most vulnerable population, the neonates, with BPZE1. This study indicates the feasibility of co-administering BPZE1 with the world’s most widely used vaccine, BCG, in this high-risk population.

### Limitations of the study

Our study has several limitations. To assess protection against *M. tuberculosis*, we infected mice by nasal route, which proved to be highly reproducible, rather than by aerosol and used a high dose to ensure that 100% of the mice were infected. This high dose has been described by De Steenwinkel et al. to mimic human TB infection in Balb/c mice.^50^ However, it would be interesting to evaluate the level of protection induced by vaccination with BPZE1 and BCG using a low (100 cfu) or ultra-low (10 cfu) challenge dose of *M. tuberculosis* delivered by aerosol.^51^

The efficacy of vaccine-induced protection was assessed over a limited period of 8–10 weeks post-vaccination. Further studies are required to evaluate the long-term durability of protection against pertussis, tuberculosis, and seasonal influenza following co-administration of BPZE1 and BCG. Additionally, our experiments were conducted in 3-week-old infant mice, without examining vaccine-induced responses in neonatal mice or assessing the potential impact of maternal immunity. All the studies were carried out with female mice. Potential gender bias will be assessed in a future study. Seasonal influenza was chosen as a model to evaluate the heterologous protection induced by BPZE1 and BCG; however, other viral and bacterial pathogens should be tested to determine the broader applicability of this heterologous protection. Finally, more in-depth analysis of the immune mechanistic pathways is needed to understand why heterologous protection induced by BCG is age-dependent, unlike the response elicited by BPZE1.

## Resource availability

### Lead contact

Requests for further information and ressources should be directed to and will be fulfilled by the lead contact Nathalie Mielcarek (nathalie.mielcarek@inserm.fr).

### Material availability

This study did not generate new unique reagents. All data reported in this paper will be shared by the lead contact upon request.

### Data and code availability

• All data associated with this study are provided in the paper or the supplemental information.
• This paper does not report original code.
• Any additional information required to reanalyze the data reported in this paper is available from the lead contact upon request.

## Acknowledgments

We thank the personnel of the animal facility of PLBS (UAR2014-US41) and the BSL-3 facility (IPL). This work was supported by Inserm, CNRS, and Institut Pasteur de Lille.

## Author contributions

Each author has contributed significantly to the work. Project design and supervision by N.M., experiments performed by C.R. and A.-S.D., data analysis by C.R., A.-S.D., S.C., and N.M., graphics drawn by C.R., literature search by C.R. and N.M., writing by C.R. and N.M., statistical analysis by S.C. and C.R. All authors have read, edited, and approved the final version of the manuscript.

## Declaration of interests

A.-S.D and N.M. hold patents on the BPZE1 vaccine, which is licensed to ILiAD Biotechnologies. N.M. is a member of ILiAD’s scientific advisory board.

## Declaration of generative AI and AI-assisted technologies in the writing process

During the preparation of this work the author(s) used DeepL in order to improve language and readability. After using this tool, the authors reviewed and edited the content as needed and take full responsibility for the content of the publication.

## STAR★Methods

### Key resources table

**Table:** 

REAGENT or RESOURCE	SOURCE	IDENTIFIER
**Antibodies**		

Goat anti-mouse IgG (H + L) HRP conjugated antibodies	Abcam	Cat# ab7068; RRID:AB_955413
Goat anti-mouse IgA HRP conjugated antibodies	Biorad	Cat# STAR137P; RRID:AB_2075637

Bacterial and virus strains		

*Bordetella pertussis* BPSM	Menozzi et al., 1994^52^	Lab collection
*Bordetella pertussis* BPZE1	Mielcarek et al., 2006^6^	Lab collection
*Bordetella pertussis* BPCTA1	Solans et al., 2018^26^	Lab collection
*Mycobacterium bovis* BCG Pasteur (1173P2)	N/A	Lab collection
*Mycobacterium tuberculosis* H37Rv	N/A	Lab collection
H3N2 influenza A virus strain Scotland/20/74	Kindly provided by F. Trottein, Institut Pasteur de Lille	N/A

**Biological samples**		

Murine lung homogenates and purified lung cells	This paper	N/A
Murine spleen homogenates and purified spleen cells	This paper	N/A
Murine nose homogenates	This paper	N/A
Murine BAL fluids	This paper	N/A
Murine sera	This paper	N/A

**Chemicals, peptides, and recombinant proteins**		

Panta antibiotic mixture	BD	245114
Thiophenecarboxylic Acid Hydrazide (TCH)	Sigma-Aldrich	T1388; CAS: 2361-27-5
KH_2_PO_4_	Sigma-Aldrich	60229; CAS: 7778-77-0
MgSO_4_	Sigma-Aldrich	M2773; CAS: 10034-99-8
L asparagine monohydrate	Sigma-Aldrich	A8381; CAS: 5794-13-8
Ammonium Iron(III) Citrate, iron 28%	Merck	3761; CAS: 1185-57-5
Citric acid monohydrate	Sigma-Aldrich	27102; CAS: 5949-29-1
ZnSO_4_ . 7H20	Sigma-Aldrich	1.08883; CAS: 7446-20-0
Tyloxapol	Sigma-Aldrich	T8761; CAS: 25301-02-4
BD BBL™ Middlebrook OADC Enrichment	Fisher Scientific	11708173
Middlebrook 7H11 agar base	Merck-Millipore	M0428
BD Difco Bordet-Gengou agar base	BD	248200
Glycerol	Euromedex	EU3550
Defibrinated sheep blood	Eurobio Scientific	SB054
Streptomycin sulfate salt	Sigma-Aldrich	S6501; CAS: 3810-74-0
Gentamycin sulfate salt	Sigma-Aldrich	G3632; CAS: 1405-41-0
Nalidixic acid sodium salt	Sigma-Aldrich	N4382; CAS: 3374-05-8
Gibco DPBS, no calcium, no magnesium	Fisher Scientific	12037539
Sodium-L(+)-glutamate monohydrate	VWR Chemicals	27872.298
L proline	Merck	81709, CAS: 147-85-3
NaCl	Sigma-Aldrich	S7653; CAS: 7647-14-5
KCl	Sigma-Aldrich	P9541; CAS: 7447-40-7
MgCl_2_ . 6H_2_O	Sigma-Aldrich	M9272; CAS: 7791-18-6
CaCl_2_ . 2H_2_O	Sigma-Aldrich	C3881; CAS: 10035-04-8
Tris base	Euromedex	EU1018-A; CAS: 77-86-1
Gibco Bacto casamino acids	Fisher Scientific	16269851
Heptakis(2,6-Di-O-Methyl)-B-cyclodextrin	MP Biomedicals	157320; CAS: 51166-71-3
L cystein	Fluka	30089; CAS: 52-90-4
Iron(II) sulfate heptahydrate	Sigma-Aldrich	F8263; CAS: 7782-63-0
Nicotinic acid	Sigma-Aldrich	N0761; CAS: 59-67-6
L-Ascorbic acid	Merck	A92902; CAS: 50-81-7
L Glutathione reduced	Sigma-Aldrich	G4251; CAS: 70-18-8
Euthasol (pentobarbital sodium 400 mg/ml)	Dechra	N/A
Atropine sulfate 1 mg/ml	Aguettant	N/A
Ketamine 1000 mg/ml	Virbac	N/A
Valium 10 mg/ 2 ml	Atnahs Pharma	N/A
EDTA	Sigma-Aldrich	E9884; CAS: 60-00-4
RPMI 1640 medium, GlutaMAX supplement	Fisher Scientific	12027599
Fetal Bovine Serum	Eurobio Scientific	CVFSVF00-01
cOmplete EDTA-free protease inhibitor cocktail	Sigma-Aldrich	5056489001
PPD 1 mg/ml	Statens Serum Institute	N/A
Tetramethylbenzidine dihydrochloride (TMB)	Interchim	664782
ConcanavalinA from *Canavalia ensiformis*	Sigma-Aldrich	C5275; CAS: 11028-71-0
Liberase TM research grade	Roche	5401127001
ACK lysis buffer	Thermo Fisher Scientific	A1049201
Percoll	GE Healthcare	17-0891601

**Critical commercial assays**		

BD OptEIA mouse IFN-γ ELISA set	Fisher Scientific	15858008
Mouse IL-17A Elisa kit	Mabtech	3521-1H-20
Mouse IL-22 uncoated ELISA kit	Invitrogen	88-7422-88
Milliplex mouse magnetic bead panel	Millipore	MCYTOMAG-70K

**Deposited data**		

Raw and analyzed data	This paper	N/A

**Experimental models: Organisms/strains**		

BALB/c mice	Charles River	N/A

**Software and algorithms**		

GraphPad Prism software version 9.3.1	GraphPad Software, San diego, CA, USA	N/A
R software version 4.1.1	R Foundation for statistical Computing, Vienna, Austria	N/A

**Other**		

Amicon Ultra-0,5 10kD	Millipore	UFC501096
Nunc MAxisorp 96-well plates	Dutscher	55133
Culture plates Microwell 96-well Nunc	Dutscher	055260
Falcon cell strainers 70 μm	Fisher scientific	352350

### Experimental model and study participant details

#### Bacterial strains

Virulent *B. pertussis* BPSM, gentamycin-resistant derivative BPCTA1, and attenuated BPZE1, a strain in which three major toxins (PT, DNT, and TCT) have been genetically inactivated or removed, were previously described.^6^^,^^26^^,^^52^ For immunization or infection, strains were grown on Bordet-Gengou (BG) agar (Difco, Detroit, Mich.) supplemented with 1% glycerol, and 20% defibrinated sheep blood. When required, 100 μg/mL streptomycin (Sigma, St. Louis, Mo.) or 10 μg/mL gentamycin (Sigma), and 30 μg/mL nalidixic acid (Sigma) were added. For the preparation of whole cell lysate, BPSM was grown in a modified Stainer Scholte liquid medium, as previously described.^53^

*M. tuberculosis* H37Rv and the BCG Pasteur strain (isolate 1173P2, World Health Organization, Stockholm, Sweden) were grown in liquid Sauton medium or on solid Middlebrook 7H11 medium (Merck).

#### Viral strain

Frozen titrated stocks of the mouse-adapted H3N2 IAV strain Scotland/20/74 were kindly provided by Dr. François Trottein, Institut Pasteur de Lille.

#### Mice

Three-week-old and 8-week-old female BALB/c mice were purchased from Charles Rivers France and were maintained under specific pathogen-free (SPF) conditions at the Institut Pasteur de Lille animal facility. Animals were housed with no more than 5 mice per cage, in 12 h light-dark cycles and had free access to food and water.

All the animal experiments were carried out in accordance with the guidelines of the French Ministry of Research on animal experiments and with institutional regulations and ethical guidelines (B59-350009, Institut Pasteur de Lille, France). The protocols were approved by the Ethical Committees of the Region Nord-Pas-de-Calais and the Ministry of Research (agreement number APAFIS # 201603311654342_v2). Experiments were conducted by qualified, accredited personnel.

### Method details

#### Vaccination and infection experiments

Depending on the experiment, the mice were vaccinated with BPZE1, BCG or both vaccines at either 3 or 8 weeks of age, as specified in the text. BPZE1 administration was performed intranasally with 1 x 10^3^ or 1 x 10^6^ bacteria in 20 μL PBS. For BCG vaccination, mice were immunized subcutaneously (s.c.) with 5 x 10^5^ to 1 x 10^6^ bacteria in 200 μL of phosphate-buffered saline (PBS).

For B. pertussis challenge infection, 2 months after vaccination, mice were intranasally (i.n.) infected with 5 x 10^6^ BPSM or BPCTA1 in 20 μL PBS. Bacterial loads in the lungs and the nose were determined 3 h or 7 days later to evaluate protective vaccine efficacy.

All infection studies with virulent *M. tuberculosis* were done in a biosafety level 3 facility, the animals being housed in isolator cages. Mice were challenged intranasally with 1 × 10^5^ CFU of H37Rv, 10 weeks after vaccination. They were sacrificed 8 weeks post-challenge. The bacterial load in the lungs and the spleen was determined by plating serial dilutions of organ homogenates on Middlebrook 7H11 medium (Merck) supplemented with 2 μg/mL 2-Thiophenecarboxylic Acid Hydrazide (TCH, 2 μg/mL, Sigma) and MGIT PANTA (BD Biosciences). The presence of TCH selectively inhibits the growth of the residual BCG bacteria. The lyophilized MGIT PANTA antibiotic mixture (polymyxin, amphotericin B, nalidixic acid, trimethoprim, azlocillin) was resuspended in OADC (BD Biosciences) and added to the culture medium at the following final concentrations: polymyxin B, 40 units/mL; amphotericin B, 4 μg/mL; nalidixic acid, 16 μg/mL; trimethoprim, 4 μg/mL; Azlocillin, 4 μg/mL. Colonies were counted after 3 weeks of incubation at 37°C.

For heterologous protection studies, mice were infected i.n. with 300 pfu of H3N2 influenza A virus in 30 μL of PBS, 6 weeks after vaccination. Five days after the challenge, five mice per group were sacrificed and bronchoalveolar lavage (BAL) fluids were recovered to analyze the local pro-inflammatory cytokine and chemokine responses. Body weight and mortality were monitored daily for 15 days post-challenge and mice were sacrificed when the body weight loss exceeded 20% of the initial weight.

All the i.n. administrations were performed on anaesthetized mice, after administration of a cocktail of ketamine, atropine and valium via intraperitoneal (i.p.) route.

#### Bronchoalveolar lavage fluid collection

Mice were lethally anesthetized with an i.p. administration of Euthasol (300 mg/kg). The trachea was cannulated and BAL fluids were recovered by two consecutive lavages with 500 and 800 μL of PBS, 2% FBS, 2mM EDTA, supplemented with a complete EDTA-free protease inhibitor cocktail (Roche). After centrifugation at 4°C and 1200 rpm for 10 min, the supernatant was recovered and stored at −80°C for subsequent cytokine analysis or antibody titration.

#### Antibody determination

Sera and BAL fluids from 5 individual mice per group were collected 21 days after immunization. BAL fluids were concentrated 25 times using Amicon Ultra-0,5 10kD (Millipore) according to the manufacturer’s specifications. IgG titer in sera as well as IgA and IgG titers in BAL were then estimated by enzyme-linked immunosorbent assays (ELISA) as previously described.^54^ Briefly, 96-well plates (Maxisorp, Nunc) were coated overnight at 4°C with 100 μL of whole BPSM cell lysate or PPD (Statens Serum Institute) resuspended in PBS at a final concentration of 10 μg/mL. BPSM lysate was prepared from a 200 mL bacterial culture grown in a modified Stainer Scholte medium. The bacteria were harvested by centrifugation, resuspended in 10 mL of PBS supplemented with a mixture of antiproteases (cOmplete mini EDTA-free Protease Inhibitor Cocktail, Roche), and lysed by passages through a French pressure cell.

Samples were added in two-fold serial dilutions and incubated overnight at 4°C. Goat anti-mouse IgG (H + L) horseradish peroxidase-conjugated antibodies (Abcam Cat# ab7068, RRID:AB_955413) or Goat anti Mouse IgA HRP conjugated antibodies (Bio-Rad Cat# STAR137P, RRID:AB_2075637) were then added for 1 h at 37°C. The ELISAs were developed using Tetramethylbenzidine dihydrochloride (TMB, Interchim) according to the manufacturer’s specifications. The results are expressed in titers, defined as the reciprocal of the dilution giving an optical density at 450 nm three times that of the conjugate control.

#### Cellular response in lungs and spleen

Lungs and spleen were harvested from individual naive or vaccinated animals, two months after immunization. Lungs were perfused with RPMI supplemented with 2% FBS, 0·5 mg/mL liberase TM (Roche), and 40 μg/mL DNase. After 30 min of incubation at 37°C, the lungs were passed through a nylon screen (Falcon cell strainer, 70 μm). Red blood cells were lysed using ACK lysis buffer (Gibco). After centrifugation for 10 min at 1200 rpm, the cell pellet was resuspended in 10 mL PBS 20% Percoll (GE Healthcare) and centrifuged at 2000 rpm without brake, at 4°C, for 12 min. One upper layer on top of the gradient containing mainly epithelial cells was removed, as well as the supernatant. The cell pellet was resuspended in RPMI 10% FBS and passed through a nylon screen again.

The splenocytes were isolated by preparing single cell suspension in RPMI medium (Gibco). After centrifugation for 8 min at 1200 rpm, the cell pellet was resuspended in ACK lysis buffer (Gibco) and incubated 5 min at 37°C in order to lyse the erythrocytes. Splenocytes were recovered by centrifugation and resuspended in RPMI medium supplemented with 10% FBS.

After counting, lung or spleen cells were distributed in microtiter wells (96-well microtiter plates, Nunc) with 5 x 10^5^ cells/well and were stimulated with 5 μg/mL whole BPSM lysate, PPD (Statens serum institute), or Concanavalin A (Sigma), or left unstimulated. After 72 h of incubation at 37°C and under 5% CO_2_, the supernatants from triplicate cultures were harvested and stored at −80°C for further analysis of the antigen-specific cellular response.

#### Cytokine analysis

The levels of IFN-γ, IL-17A and IL-22 in culture supernatants of *in vitro* restimulated lung and spleen cells were determined using the following immunoassay kits: BD OptEIA mouse IFN-γ ELISA set (Pharmingen), mouse IL-17A Elisa kit (Mabtech), mouse IL-22 uncoated Elisa kit (Invitrogen).

Cytokines and chemokines were quantified in BAL fluids, 5 days after a heterologous challenge with H3N2 influenza A virus, using a Luminex assay with a Milliplex mouse magnetic bead panel (Merck, Millipore). The following cytokines were quantified IL-1β, IL-4, IL-6, IL-10, IL-17A, IL-22, IFN-γ, TNF, and the following chemokines: CCL2/MCP-1, CCL3/MIP-1α, CCL4/MIP-1β, CCL5/RANTES, CXCL2/MIP-2, and CXCL10/IP-10.

### Quantification and statistical analysis

To analyze the protective efficacy of the vaccines against a virulent *B. pertussis* challenge, a two-factor Aligned Rank Transform (ART) ANOVA was applied followed by a Conover post-hoc analysis (Bonferroni-adjusted *p*-values) for pairwise comparisons.^55^ Mycobacterial load, antibody titers, as well as cytokine levels were analyzed using a Kruskal-Wallis test followed by a Conover post-hoc test for pairwise and many-to-one comparisons (Bonferroni-adjusted *p*-values). For heterologous protection experiments, mixed-effects models were used to compare weight loss over time between different conditions. The *log*-*rank test* was used for survival analysis. Heatmaps were generated to visualize the standardized data, where Z-scores were computed row-wise. The Z-score transformation was applied to center the data around zero and normalize it to unit variance, thereby enabling direct comparisons across rows.^56^ All the graphs, calculations, and statistical analyses were performed by using GraphPad Prism software version 9.3.1 (GraphPad Software, San Diego, CA, USA) and R software version 4.1.1 (R Foundation for Statistical Computing, Vienna, Austria).

∗, *p* ≤ 0.05; ∗∗, p ≤ 0.01; ∗∗∗, p ≤ 0.001 and ∗∗∗∗, p ≤ 0.0001.
