# Supplementary material for: Protection of infant mice against pertussis, tuberculosis and influenza by co-administration of nasal pertussis vaccine candidate BPZE1 and BCG
Source: iScience. 2025 Jun 7;28(7):112839. doi: 10.1016/j.isci.2025.112839 (PMC12221715; doi:10.1016/j.isci.2025.112839)
Supplement: Document S1. Figures S1 and S2 [file mmc1.pdf]

iScience, Volume *28*

Supplemental information

Protection of infant mice against pertussis, tuberculosis and influenza by co-administration of nasal pertussis vaccine candidate BPZE1 and BCG

Carine Rouanet, Anne-Sophie Debrie, Stephane Cauchi, and Nathalie Mielcarek

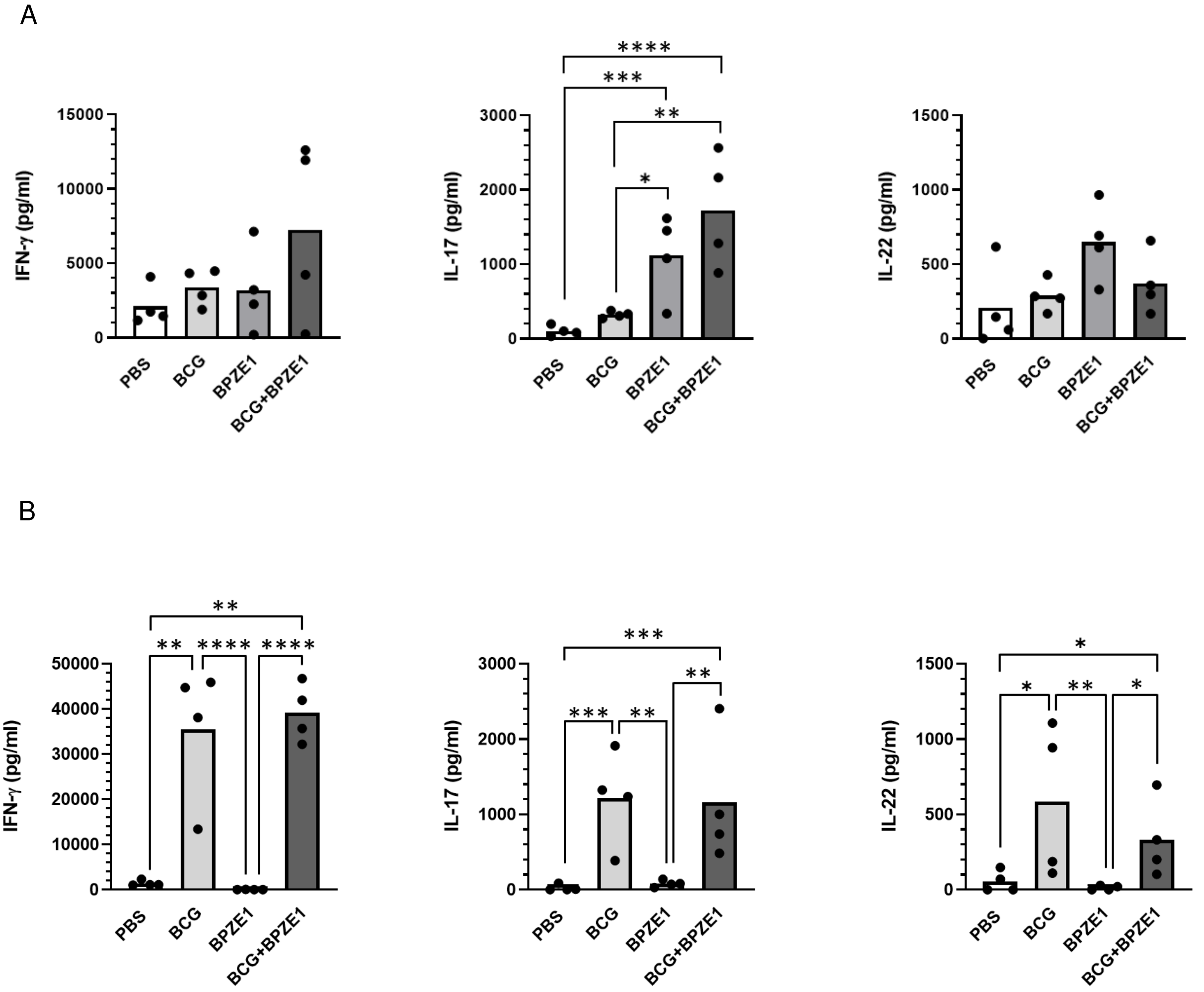

Figure S1. Specific cellular immune response in the spleen after BPZE1 and/or BCG vaccination, Related to Figure 3. Infant mice were immunized with BCG s.c., BPZE1 i.n., or both vaccines simultaneously. Two months after vaccination, mice were sacrificed and splenocytes were stimulated with *B. pertussis* lysate (A) or PPD (B). The secretion of IFN-γ, IL-17A, and IL-22 in the culture supernatant was quantified by ELISA. The results are expressed as individual values and mean for four mice per group. A Kruskal-Wallis test followed by a Conover post-test was used to determine statistical significance. *p*-values are defined as ≤ 0.05, ∗; ≤ 0.01, ∗∗; ≤ 0.001, ∗∗∗; ≤ 0.0001,∗∗∗∗.

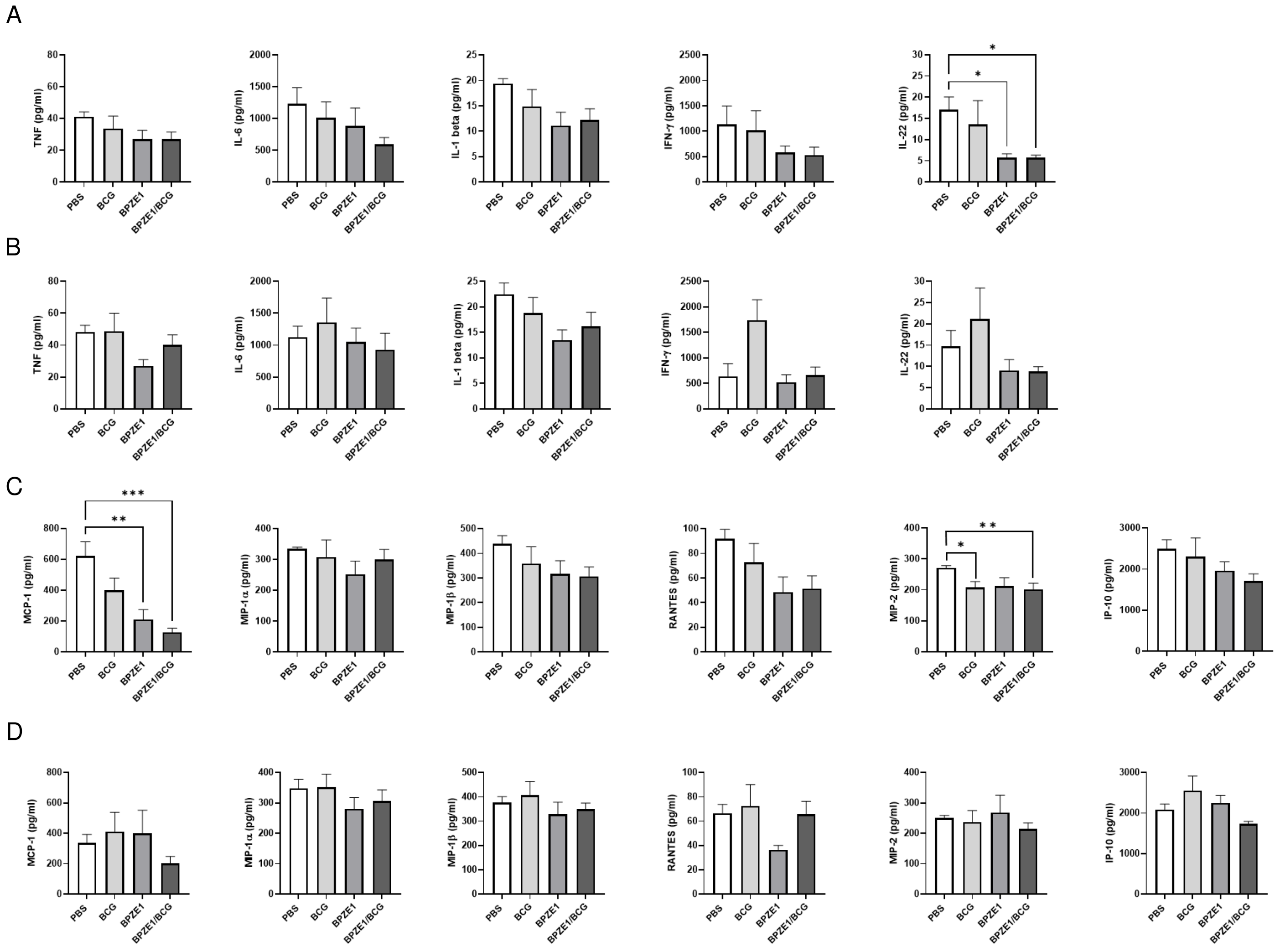

Figure S2. Cytokine and chemokine production, in murine BAL fluids, 5 days after challenge with influenza A virus, Related to Figure 6. Infant (A, C) or adult BALB/c (B, D) mice were vaccinated with BCG s.c., BPZE1 i.n., or both vaccines simultaneously. Viral challenge was performed six weeks after vaccination. Five days after the infection, the animals were sacrificed and BAL fluids were collected. The production of various cytokines (A, B) and chemokines (C, D) was quantified by Luminex in the individual BAL fluid samples. Results are expressed as mean +/-SEM from five mice per group. A Kruskal-Wallis test followed by a Conover post-test was used to determine statistical significance. *p*-values are defined as ≤ 0.05, ∗; ≤ 0.01, ∗∗.
